# Use of thin-layer chromatography to detect counterfeit sulfadoxine/pyrimethamine tablets with the wrong active ingredient in Malawi

**DOI:** 10.1186/s12936-016-1259-9

**Published:** 2016-04-14

**Authors:** Felix Khuluza, Stephen Kigera, Richard W. O. Jähnke, Lutz Heide

**Affiliations:** Pharmacy Department, College of Medicine, University of Malawi, Private Bag 360, Chichiri, Blantyre 3, Malawi; Mission for Essential Drugs and Supplies (MEDS), P.O. Box 78040-00507, Viwandani, Nairobi, Kenya; Global Pharma Health Fund e.V. (GPHF), Rotlintstraße 75, 60389 Frankfurt, Germany; Presented address: Pharmaceutical Institute, Eberhard-Karls-University Tuebingen, Auf der Morgenstelle 8, 72076 Tuebingen, Germany

**Keywords:** Falsified medicines, SSFFC medicinal products, Sulfadoxine/pyrimethamine, Thin-layer chromatography, GPHF Minilab

## Abstract

**Background:**

Substandard and falsified anti-malarial medicines pose a serious threat to public health, especially in low-income countries. Appropriate technologies for drug quality analysis in resource-limited settings are important for the surveillance of the formal and informal drug market. The feasibility of thin-layer chromatography (TLC) with different solvent systems was tested using the GPHF Minilab in a study of the quality of sulfadoxine/pyrimethamine tablets in Malawi.

**Methods:**

Twenty eight samples of sulfadoxine/pyrimethamine tablets were collected from randomly selected health facilities of four districts of southern Malawi. A mystery shopper approach was used when collecting samples from illegal street vendors, and an overt approach for the other facilities. Samples were subjected to visual inspection, disintegration testing and TLC analysis. 10 samples were further investigated according to the methods of the US Pharmacopeia using high performance liquid chromatography (HPLC).

**Results:**

One sample was found to be falsified, containing a mixture of paracetamol tablets and co-trimoxazole tablets. These had been repackaged into paper strip packs labelled as a brand of sulfadoxine/pyrimethamine. TLC with different solvent systems readily proved that these tablets did not comply with their declaration, and provided strong evidence for the active pharmaceutical ingredients which were actually contained. Full pharmacopeial analysis by HPLC confirmed the results suggested by TLC for this sample, and showed two further samples to be of substandard quality.

**Conclusions:**

Due to the absence of the declared anti-malarial ingredients and due to the presence of other pharmaceutical ingredients, the identified falsified medicine represents a serious health risk for the population. Thin-layer chromatography (TLC) using different solvent systems proved to be a powerful method for the identification of this type of counterfeiting, presenting a simple and affordable technology for use in resource-limited settings.

## Background

Substandard and falsified antimalarial medicines pose a serious threat to public health. The worldwide spread of falsified medicines has been addressed as a “global pandemic”, and in this context it has been correctly emphasized that “diagnostics are at the heart of any successful epidemic response effort” [1]. Therefore, analytical methods to identify falsified medicines are essential in order to fight this specific pandemic.

The gold standard methods for drug quality analysis are defined in the leading pharmacopeias, such as the International Pharmacopeia, the United States Pharmacopeia, the British Pharmacopeia, the Pharmacopeia of Japan, and others. They rely primarily on high performance liquid chromatography (HPLC) for analysis of the content of the active ingredient, of dissolution, and of the presence of related substances. The required instruments cost 50,000–100,000 $ (USA) for standard equipment, and more for advanced equipment [2]. They are complicated and delicate mechanical and electronic tools, requiring careful handling by trained professionals as well as an infrastructure including an electricity supply of constant voltage, very pure organic solvents, and regular maintenance by skilled technicians. For low-income countries, the full pharmacopeial analysis of medicines is a formidable challenge and can usually be achieved only for a limited number of samples in the national drug quality control laboratories. In most cases, no capacity exists in such countries for the regular surveillance of drug quality on the various levels of the drug supply chain, thereby opening the possibility for substandard and falsified medicines to enter the market [3].

Appropriate technologies for drug analysis in resource-limited settings would allow a more regular surveillance of the formal and informal drug market, aiding in the rapid detection of falsified medicines and potentially deterring criminal counterfeiters from bringing their products into the market. A number of such appropriate analytical technologies have been reported [2–4]. Few of them are ready for wide-spread application. The best-established one is thin-layer chromatography (TLC). For drug quality analysis in resource-limited settings, TLC is mostly employed in form of the Minilab™ supplied by the Global Pharma Health Fund (GPHF), a charity supported by the Merck pharmaceutical company [5]. The Minilab is a pre-assembled kit containing all analytical tools for the qualitative and semi-quantitative TLC analysis of about 100 essential medicines, and does not require electricity, running water or any sophisticated infrastructure. It is supplied with a manual describing the analytical procedure for each drug, and only very limited training is required for its use. TLC analysis using the Minilab has been used in many studies in Africa, Asia and South America [6, 7]. However, also limitations of this technology in comparison to full pharmacopeial analysis have been pointed out [8].

Before embarking on a larger study on the quality of antimalarial drugs in Malawi, the feasibility of TLC analysis was tested using samples of the anti-malarial drug sulfadoxine/pyrimethamine (SP) as example. In governmental and church health facilities of Malawi, SP is used for intermittent preventive malaria treatment in pregnancy. Private vendors frequently sell SP also to other patients as a single-dose malaria therapy, despite the fact that artemisinin-based combination therapies are now recommended as first-line therapy for malaria. In the course of this pilot study, a falsified SP sample was identified which contained active ingredients different from the declared ones. TLC analysis using different solvent systems allowed not only to prove that this sample did not conform to its declaration, but also provided strong evidence which active ingredients were actually contained. This demonstrates a power and versatility of TLC analysis which should be considered when the relative merits of different analytical techniques for drug quality analysis are discussed. This study highlights the usefulness of TLC analysis especially in the case of falsified drugs which contain active ingredients different from the declared ones.

## Methods

### Sample collection

Out of the 13 districts of southern Malawi, four were randomly selected. From each of these districts, a list of government health centers was obtained. From each of three districts, two health centres were selected randomly, and samples were collected from these health centres as well as from the respective district hospital. The fourth district comprised one of Malawi’s larger cities. From this district two urban and two rural health centres were randomly selected, and samples were collected from there, from the district health office and from the central hospital. If church-affiliated health facilities, private pharmacy shops, drug stores, or illegal street vendors could be identified nearby the selected government health facilities, then drugs samples were also collected from there. Samples were collected by members of the Pharmacy Department, University of Malawi. A mystery shopper approach was used for the illegal street vendors, and an overt approach for the other facilities. The mystery shopper stated that he had been asked by friends in his village to buy this medicine for them. Samples of 150 tablets were collected if available, otherwise smaller numbers. If medicines with generic and brand name were available, the brand name medicine was sampled. If several brand name medicines were available, the most expensive brand name medicine was sampled. In most sites, however, only a single type of sulfadoxine/pyrimethamine tablets was available. Samples were transported to the Pharmacy Department, College of Medicine, Blantyre, within 48 h, and stored below 25 °C until analysis.

### Visual inspection, disintegration testing and testing for uniformity of mass of dosage units

The external packaging, primary packaging and (if available) package leaflets were inspected, including batch number and expiry dates. The tablets were visually inspected, especially for undamaged, unaltered surfaces and colour uniformity. Disintegration testing for instant-release oral dosage forms was carried out according to the manual of the GPHF Minilab [9]; in short, six tablets were kept in water at 37 °C under occasional shaking or stirring, and complete disintegration within 30 min was confirmed. For uniformity of mass of dosage unit, the exact weight of 20 tablets was determined; acceptable deviations were: up to ±5 % in at least 18 tablets, and up to ±10 % in no more than two tablets.

### Thin-layer chromatographic (TLC) testing

TLC testing was done according to the procedure given by the manual of the GPHF Minilab for sulfadoxine (including SP formulations) [9]. From each sample, three tablets were analysed individually. In short, each tablet was crushed to a fine powder and extracted with 20 ml methanol by vigorous shaking for 3 min. After sedimentation of undissolved residues, 1 ml of the supernatant was removed and diluted with 3 ml methanol. Using a microcapillary, 2 µl of this solution were applied to a TLC plate (Merck silica gel 60 F254, 0.2 mm thickness, 5 × 10 cm). Authentic standard solutions of sulfadoxine/pyrimethamine with known concentrations were applied as comparison. The plate was developed in a solvent system of ethyl acetate:methanol 15:5 for approximately 15 min. After drying off the residual solvent, the active pharmaceutical ingredients were visualized first under UV light (254 nm), and subsequently by iodine vapour. The results were documented using an inexpensive digital camera (Canon PowerShot SX600 HS).

For comparison to authentic paracetamol and co-trimoxazole samples, the solvent systems given by the manual of the GPHF Minilab for analysis of paracetamol[9], pyrimethamine [10] and co-trimoxazole [9] were used, i.e. acetone:toluene:acetic acid 10:10:0.5 (for the experiment depicted in Fig. 2b); ethyl acetate:methanol:acetone:conc.aqueous ammonia 12:6:2:0.5 (Fig. 2c); ethyl acetate:methanol 15:5 (Fig. 2d).

### HPLC analysis according to the United States Pharmacopeia (USP)

Following the methods specified in USP38-NF33, identification of the active ingredients by TLC and HPLC, HPLC analysis (=assay) for the content of sulfadoxine and pyrimethamine, analysis for uniformity of dosage units with respect to the content of the active ingredients, and analysis of their dissolution was carried out in the WHO-prequalified drug quality control laboratory of the Mission for Essential Drugs and Supplies, Nairobi, Kenya. HPLC analysis for sulfadoxine and pyrimethamine was carried out using a Gemini 5 µm C6-Phenyl 110Å HPLC column 250 × 4.6 mm (Phenomenex, USA) and an isocratic solvent system of 0.1 % aqueous phosphoric acid:acetonitrile 83:17, flow rate 1.2 ml/min. The wavelength for UV detection was 230 nm. For the identification of paracetamol and co-trimoxazole, the respective methods of USP38-NF33 for those drugs were used.

### Ethical approval

This study was approved by the College of Medicine Research and Ethics Committee, University of Malawi.

## Results

### Sample collection

Twenty eight samples of sulfadoxine 500 mg/pyrimethamine 25 mg tablets were collected in four districts in southern Malawi. 15 were collected from government health facilities, seven from church-affiliated health facilities, four from private pharmacies and drug stores, and two from illegal street vendors. 21 of the samples were found to be distributed under the generic name “sulfadoxine/pyrimethamine”, and seven under brand names. According to the information on the packaging, the samples had been produced by six different manufacturers, with 18 samples produced in in India (by two different manufactures), three in China, three in Tanzania, two in Cyprus and two in Malawi. Comparison with the records of the Pharmacy, Medicines and Poisons Board, i.e. the national drug regulatory agency of Malawi, showed that the SP tablets from five of the manufacturers were registered in Malawi, but the tablets from one of the manufactures were not. The non-registered type represented the most common SP preparation collected in government and church-affiliated health facilities, accounting for 17 of the 28 samples.

### Visual inspection

**Fig. 1:**
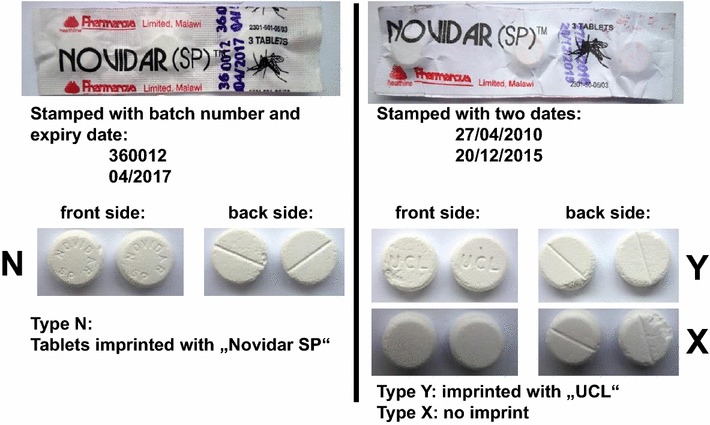
Primary packaging and tablets of Novidar (SP)™ and of the falsified samples type X and type Y

Only a single sample clearly failed visual inspection. It was purchased from an illegal street vendor and was sold in an opened, apparently genuine cardboard box labelled “Novidar (SP)”, a brand name of SP manufactured and sold by the Malawian manufacturer Pharmanova Ltd. The cardboard box contained paper strip packs labelled “Novidar (SP)” which, however, were found to be of two different kinds (Fig. 1). One (hereafter called type N) was stamped with the same batch number and expiry date as given on the outer packaging (i.e. the cardboard box). The other one was stamped with two dates (“27/04/2010” and “20/11/2015”), different from those given on the outer package. These paper strip packs were made from a thinner type of paper than those of type N. A part of the tablets in these strip packs had apparently adsorbed moisture. Upon opening of the strip packs, some tablets were found to stick to the paper, and to break easily.

Fifteen paper strips of this kind were contained in this sample. Although they were all uniform in their appearance and stamp, they were found to contain two different kinds of tablets (Fig. 1). One (hereafter called type X) did not carry an imprint on its front side. The other one (hereafter called type Y) was imprinted with the letters “UCL”. In contrast, the tablets with the paper strips of type N were imprinted with “Novidar SP” on the front side, which is consistent with the genuine product of Pharmanova Ltd.

### Thin-layer chromatographic analysis

**Fig. 2:**
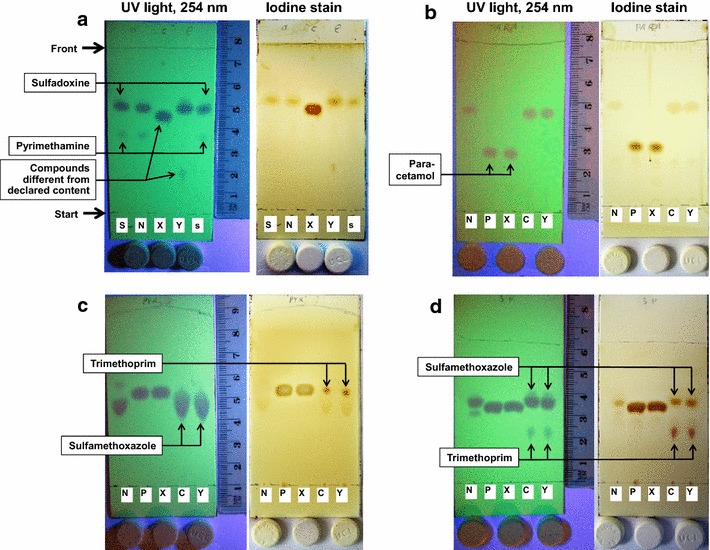
TLC analysis of Novidar (SP)™ tablets (labled as N) and of the falsified samples type X and type Y (labeled as X and Y). **a** Comparison to an authentic standard of sulfadoxine 500 mg/pyrimethamine 25 mg (*S*) and to an authentic standard containing only 80 % of these amounts (*s*). **b**–**d** Comparison to paracetamol 500 mg (*P*) and co-trimoxaxole 480 mg (*C*), using different TLC solvent systems

The tablets of types N, X and Y were subjected to thin-layer chromatographic (TLC) analysis according to the procedure given in the manual of the GPHF Minilab for sulfadoxine/pyrimethamine tablets (see Methods section). An authentic standard of sulfadoxine 500 mg/pyrimethamine 25 mg was used for comparison. Detection was carried out first with UV light (254 nm), then with iodine staining. The result is shown in Fig. 2a. The complete analytical procedure was repeated again, starting from different tablets. The results were identical to those shown in Fig. 2a.

Both in the first and the second analysis, the tablets of type N showed spots identical in Rf value and intensity to those of sulfadoxine and pyrimethamine in the authentic standard. This strongly indicates that this product contains the declared active ingredients in the declared amounts, and most likely represents the original product “Novidar (SP)” of the Malawian manufacturer Pharmanova Ltd. Type X showed no spots identical in Rf value to those of sulfadoxine and pyrimethamine. This proves the absence of relevant quantities of both active principles in this product. Instead, TLC analysis showed another compound, giving a spot of an Rf value slightly lower than sulfadoxine. The different Rf value, and the different response to iodine staining (Fig. 2a), prove that this compound is different from sulfadoxine.

Type Y showed no spot identical in Rf value to that of pyrimethamine from the authentic standard. This proves the absence of relevant quantities of pyrimethamine in this product. However, TLC analysis did show a spot similar in Rf value and intensity to that of sulfadoxine, indicating the presence either of sulfadoxine or of a compound with similar chromatographic behaviour. Furthermore, TLC analysis showed a spot of a further compound, with an Rf value clearly lower than pyrimethamine.

The imprint “UCL” is used by the Kenyan pharmaceutical manufacturer Universal Corporations Ltd. Therefore, tablets of type Y were compared the with drugs from this manufacturer. And indeed, Sulfran™ tablets (co-trimoxazole 480 mg) marketed by UCL in Malawi were found to be perfectly identical in shape, size and imprint to the tablets of type Y. It was furthermore speculated that type X may represent paracetamol tablets.

To test these hypotheses, the tablets were further analyesed in comparison to authentic paracetamol 500 mg tablets and to co-trimoxazole 480 mg tablets using the TLC solvent systems given in the manual of the GPHF Minilab for paracetamol (Fig. 2b) for pyrimethamine (Fig. 2c) and for sulfamethoxazole and co-trimoxazole (Fig. 2d). In all three solvent systems, type X showed identical results as paracetamol 500 mg tablets, and type Y showed identical results as co-trimoxazole 480 mg. Both type X and type Y proved to be clearly different from their declared content, i.e. sulfadoxine/pyrimethamine.

### Visual inspection, TLC analysis and disintegration testing of further samples

Of the 27 further SP samples collected in this study, one showed chippings upon visual inspection (hereafter called sample Z). Otherwise, all samples passed visual inspection, as well as TLC analysis and disintegration testing, performed according to the Minilab manual, and also testing of the uniformity of mass of dosage units.

### Full pharmacopeial analysis including High-Performance Liquid Chromatography (HPLC)

Ten samples were subjected to a full pharmacopeial analysis according to the methods of the United States Pharmacopeia (USP) in the WHO-prequalified drug quality control laboratory of the Mission for Essential Drugs and Supplies (MEDS) in Nairobi, Kenya. These included the sample containing a mixture of tablets of types X and Y, the sample Z showing chippings, and eight further, randomly selected samples. While authentic sulfadoxine and pyrimethamine standards showed HPLC retention times of 8.24 and 3.26 min, respectively, tablets of type X showed a peak at 3.43 min (paracetamol), and tablets of type Y showed peaks at 9.07 min (sulfamethoxazole) and 2.11 min (trimethoprim), proving that these tablets did not contain the declared active ingredients. Using authentic standards for paracetamol and co-trimoxazole and the appropriated USP methods for these drugs, the tablets of type X and type Y were confirmed to represent paracetamol 500 mg tablets and co-trimoxazole 480 mg tablets.

The sample with chippings (sample Z) failed pharmacopeial analysis both for sulfadoxine content (71.8 % of declared content) and for dissolution of sulfadoxine and pyrimethamine (55.5 % and 52 % dissolution in 30 min). One further sample failed dissolution for pyrimethamine (37.6 % dissolution in 30 min). Of the total of 10 samples subjected to analysis according to the USP, therefore seven passed the analysis in all aspects.

## Discussion

This study identified a sample labeled as sulfadoxine 500 mg/pyrimethamine 25 mg tablets which was sold by an illegal street vendor in Malawi and which contained, instead of the declared content, a mixture of paracetamol 500 mg tablets and co-trimoxazole 480 mg tablets. Apparently, paracetamol and co-trimoxazole tablets had been intentionally mislabelled for reasons of profit. In the International Drug Price Indicator Guide 2014 [11], the prices of one tablet of paracetamol 500 mg and co-trimoxazole 480 mg are given as 0.48 and 1.26 US cents, respectively, in international bulk procurement. In contrast, the price of sulfadoxine 500 mg/pyrimethamine 25 mg is given as 7.17 US cents. A similar difference exists in the retail prices of these medications in Malawi. In the price list of the Medical Aid Society of Malawi (MASM), they are given as 15, 20 and 80 Malawi Kwacha, corresponding to 2.60, 3.47 and 13.9 US cents per tablet, respectively (John Mponda, MASM, personal communication).

Due to the absence of the declared anti-malarial ingredients, and due to the presence of other pharmaceutical ingredients with their own potential risks and adverse effects, these falsified medicines represent a serious health risk for the population. The national drug regulatory agency, i.e. the Pharmacy, Medicines and Poisons Board of Malawi (PMPB) was informed about this finding.

For poor-quality and falsified medicines there is yet no universally accepted terminology. The World Health Organization (WHO) summarily addresses them as “substandard/spurious/falsely-labelled/falsified/counterfeit (SSFFC)” medicinal products. Several authors classify them into three main categories [1]: (1) falsified medicines, resulting from intentional fraudulent manufacturing; (2) substandard medicines, resulting from unintentional errors caused in manufacturing; and (3) degraded medicines which become of poor quality due to poor storage or transport conditions, or to poor handling. Two samples in this study failed pharmacopeial analysis, both due to insufficient dissolution and one also for insufficient content of an active ingredient. They may belong to the second or third category mentioned above. Only one of them (sample Z) showed defects already in visual inspection, but both passed TLC analysis and disintegration testing following the procedures of the GPHF Minilab manual [9]. This is consistent with earlier results that full pharmacopeial analysis is required for reliable detection of substandard or degraded medicines [8].

Falsified medicines, the first category mentioned above, may be further subdivided according to their composition and the resulting risk which they pose for public health: (a) falsified medicines which contain the declared active ingredients and are of acceptable quality; (b) falsified medicines which contain insufficient amounts of active ingredient or are of insufficient quality; (c) falsified medicines which contain no active ingredient; (d) falsified medicines which contain other active ingredient than the declared ones. The latter category presents the highest threat to public health. The present finding of an SP sample in Malawi which contains not the declared active pharmaceutical ingredients but different ones falls into this category. For small gangs of criminals the procedure of misappropriating drugs, relabelling them as more expensive medicines and selling them with higher profit may become increasingly attractive: since there are no production costs other than for the repackaging, this procedure probably offers a higher profit margin than any other method of drug counterfeiting. Therefore, widespread surveillance for that kind of counterfeiting may be desirable, especially in poor countries where this type is most likely to occur.

The TLC experiments using different solvent systems shown in Fig. 2 show the power and versatility of thin-layer chromatography in the identification of falsified medicines which contain the wrong active ingredient. Using only very simple equipment and inexpensive chemicals, these experiments not only proved that the investigated samples of type X and Y did not conform to their declaration of sulfadoxine/pyrimethamine, but also provided strong evidence that they actually represented paracetamol and co-trimoxazole tablets, respectively. To the best of the knowledge of the authors, no other readily available analytical technology could have given this result with comparable cost, speed and ease.

Obviously, the simple and inexpensive TLC technology has limitations. Figure 2 shows that TLC analysis could not differentiate between the chemically quite similar compounds sulfadoxine and sulfamethoxazole. In contrast, the higher resolution power of HPLC was able to differentiate between these compounds, showing retention times of 8.24 and 9.07 min, respectively.

## Conclusions

Out of 28 samples of sulfadoxine/pyrimethamine tablets collected in Malawi, one was found not to contain the declared active ingredients but to represent a mixture of paracetamol and co-trimoxazole tablets. This type of counterfeiting represents a serious risk to public health. Thin-layer chromatography (TLC) using different solvent systems proved to be a powerful, affordable and simple method for the identification of this sample, presenting an appropriate technology for drug analysis in resource-limited settings.
